# The Association Between Level of Physical Activity and Body Mass Index, and Quality of Life Among Elderly Women

**DOI:** 10.3389/fpsyg.2021.804449

**Published:** 2021-12-23

**Authors:** Anđela Ðošić, Danijela Živković, Zoran Milanović, Mladen Živković, Ljiljana Bjelaković, Marija Bratić, Saša Pantelić

**Affiliations:** ^1^Faculty of Sport and Physical Education, University of Niš, Niš, Serbia; ^2^Science and Research Centre Koper, Institute for Kinesiology Research, Koper, Slovenia; ^3^Faculty of Sports Studies, Incubator of Kinanthropological Research, Masaryk University, Brno, Czechia; ^4^Faculty of Science and Mathematics, University of Niš, Niš, Serbia

**Keywords:** physical activity, health-related quality of life, elderly, body mass index, obesity

## Abstract

The process of aging leads to changes in functional abilities, fitness levels, level of physical activity, and body mass index (BMI), all of which causes changes in the quality of life. The current study aims to determine the association between the level of physical activity (PA) and BMI, and quality of life (QoL) among elderly women. The total sample numbered 156 women, with an average age of 67.7 ± 5.6 years. To determine the level of physical activity, the self-reported International Physical Activity Questionnaire Long Form (IPAQ-LF), was used. To calculate the values of the BMI, the standard procedure recommended by the World Health Organization was used. Quality of life was evaluated using the short form of the WHOQOL-BREF questionnaire designed by the World Health Organization. All the data were processed using the statistical package for data analysis SPSS 20.0. Pearson’s correlation analysis shows statistically significant relations at the level of *p* < 0.01 between PA and Physical health in Housework = 0.36, Leisure time PA = 0.27, Walking = 0.24, Moderate PA 0.43, Total PA = 0.43, while the correlations between the variables at the significance level of *p* < 0.05 were determined in PA in transportation = 0.19 and High-intensity PA = 0.16. Multiple linear regression analysis of different levels of PA as independent variables on individual domains of QoL shows that there is an association of PA and Physical health (Sig = 0.000), more precisely, Total PA is statistically the most closely related to Physical health (Sig = 0.000), and then follows PA at work and Social relations (Sig = 0.036). Similar results were obtained when BMI is added to model A. In contrast to model A, model B shows a statistically significant association between PA and BMI with the environmental domain of QoL (Sig = 0.001). The results of the current study indicate that high- and moderate-intensity physical activity both have benefits for physical health, whereas moderate PA showed higher significance levels. Results also show that elderly women with higher BMI values achieve better results in the environmental domain of QoL.

## Introduction

The aging process is associated with physiological, functional and fitness changes in the human body which could impact everyday life of the individual. Previous studies confirmed that aging is related to lower functional abilities (Izquierdo and Cadore, 2014), muscle power (Ramirez-Campillo et al., 2016) and balance, which leads to a decrease in quality of life and an increase in the risk of various illnesses (Brown et al., 2012). It is well known that elderly people are less physically active compared to the adults (Tomioka et al., 2011) which could have many consequences in terms of health. Several studies confirmed that higher level of physical activity is associated with decreasing morbidity and mortality as well as increased quality of life (Brown et al., 2012; Arem et al., 2015; Ekelund et al., 2019; Kraus et al., 2019). However, the concurrent effect of physical activity and body mass index (BMI) on the quality of life in elderly women is still unclear due to quality of life complexity.

It is well known that active lifestyle leads to better results when it comes to mental health (Bernard et al., 2018), social functioning (Ku et al., 2016) and emotional roles (Delle Fave et al., 2018). Moreover, physical activity benefits have been confirmed in physical performances, psychological status, social relations, and the environment (Valenti et al., 2008), as well as physical health (Battaglia et al., 2016; Nawrocka et al., 2019). In line with that, Ekwall et al. (2009) confirmed that elderly women who regularly take part in physical activity have a better quality of life (Ekwall et al., 2009). Furthermore, better quality of life also depends on different intensities of physical activity and the frequency of exercise, both in the domains of physical and mental components (Dugan et al., 2009; Ekwall et al., 2009). Women who take part in high-intensity physical activity with increased energy consumption (>1,000 kcal/week) achieve better results for quality of life in almost all domains, primarily in emotional roles, vitality, and in the domain of mental health (Morimoto et al., 2006). Improved results for quality of life in the physical and psychological domain and the domain of the environment are also achieved by women who take part in moderate-intensity physical activity (Fox et al., 2007). Despite the fact that both high- and moderate-intensity physical activity increase the quality of life it is still questionable if elderly people with higher BMI achieve better quality of life if they perform high-intensity physical activity.

In addition to the connection between physical activity and quality of life, data indicate that there is also a certain association between BMI and quality of life (Søltoft et al., 2009; McDonough et al., 2013; Liu et al., 2016; Kolotkin and Andersen, 2017; You et al., 2018). Existing research indicates that BMI has an impact on almost all the aspects of quality of life. When it comes to the values of BMI, maximum quality of life is noted for women with BMI around 24.5 kg/m^2^ (Søltoft et al., 2009). This indicates that higher BMI is in a negative relation with the quality of life of obese individuals (McDonough et al., 2013) but little is known how different physical activity levels in combination with higher BMI impact quality of life.

Some of the aforementioned studies separately focused on the association between physical activity and quality of life among the elderly (Morimoto et al., 2006; Dugan et al., 2009; Ekwall et al., 2009; Bernard et al., 2018; Delle Fave et al., 2018; Nawrocka et al., 2019; Schultchen et al., 2019), while a certain number of studies investigated the association between BMI and quality of life among the same population (Søltoft et al., 2009; McDonough et al., 2013; Liu et al., 2016; Song et al., 2016; Busutil et al., 2017; Kolotkin and Andersen, 2017; You et al., 2018). However, many elderly women have been classified as overweight or obese based on BMI despite optimal physical activity level because BMI is an approximate measure that does not take into consideration body fat distribution. Therefore, physical activity and BMI should be combined as related measures to better understand their effects on quality of life as well as which measure has a higher impact. However, the full extent to which the concurrent effect of physical activity and BMI is associated with quality of life in the elderly is uncertain due to limited evidence in current literature. Therefore, the study aimed to determine the association between physical activity and BMI, and quality of life among elderly women.

## Materials and Methods

### Study Design and Procedures

The study included a questionnaire on the level of physical activity and quality of life which was filled out by elderly women. Prior to the completion of the questionnaire, the researchers provided additional explanations and instructions for the respondents regarding the way the survey questionnaire was to be completed, as well as regarding the aim and goals of the study. The respondents wrote their responses directly into the survey questionnaire, and no time limit was imposed. In ensure honesty and lack of bias when filling out the questionnaire, the respondents were informed that their responses would remain anonymous, and that the obtained results would only be used for research purposes. Incomplete questionnaires were not included in further analyses. Body mass and body height were measured following the completion of the questionnaire. Of the 194 completed survey questionnaires, 156 met the requirements for further analysis and were included in the statistical data analysis. The questionnaire also included sociodemographic questions. The research was approved by the Ethics Committee of the Faculty of Sport and Physical Education, University of Niš. All of the procedures during the study were carried out in accordance with the Declaration of Helsinki.

### Participants

The population from which the sample of respondents was extracted was defined as the population of elderly women based on criteria provided by the World Health Organization (Papalia et al., 1992). The total sample consisted of 156 elderly women recruited from the region of Southern and Eastern Serbia (one of the five Serbian regions). The average age of the respondents was 67.74 ± 5.68 years, body height 164.47 ± 6.08 cm, and body mass 69.56 ± 11.43 kg. The study included only those subjects who were able to care for themselves independently, i.e., independently perform daily tasks (dressing, bathing, feeding, walking, going to the store, etc.). Including criterion was also that subjects did not have cognitive impairments, mental disorders or dementia. The study did not include respondents with physical disabilities and severe medical conditions not related solely to the aging process (for example, malignant tumors, dementia, Alzheimer’s, etc.) or respondents who have psychological disorders. Those respondents who were in the phase of recovery from acute diseases, as well as people with visual and hearing impairments were also excluded. Subjects with cardiovascular disorders identified through medical history or when completing questionnaires were also excluded from the study.

All of the respondents were fully informed about the study procedures and were told that they could at any point opt out of the study.

### Measures

#### Physical Activity

To determine the level of physical activity, the self-reported International Physical Activity Questionnaire Long Form (IPAQ-LF) was used (Craig et al., 2003). This questionnaire evaluates the level of physical activity performed during the course of 1 week. The IPAQ questionnaire evaluates the frequency, duration, and intensity of physical activity in several domains, including: (a) leisure time physical activity, (b) physical activity around the house and garden (yard), (c) physical activity at work, and (d) transport-related physical activity. The results related to certain domains required that the points for walking, moderate physical activity, and high-intensity physical activity as part of every individual domain be added up, while the results related to certain activities required the summation of results for specific types of activities in a number of domains. The criteria for a certain level of physical activity were determined once it was taken into consideration that the IPAQ questionnaire includes questions that refer to the domain of everyday life. This results in average values for the estimation of MET-minutes. The long-form version of the IPAQ questionnaire proved to have a more acceptable level of reliability and validity (Craig et al., 2003; Tomioka et al., 2011). The reliability of the Serbian version of the IPAQ questionnaire was confirmed in the study of Milanović et al. (2014).

#### BMI

To calculate the values of BMI, the standard procedure involving the equation BMI = body mass (kg)/body height^2^ (m^2^) was used (World Health Organization, 1997).

#### Quality of Life

To evaluate quality of life, the short form of the WHO WHOQOL-BREF questionnaire for the evaluation of quality of life was used (Berlim et al., 2005). This questionnaire was developed by the World Health Organization based on the WHOQOL-100 questionnaire (WHOQOL Group, 1994; Szabo, 1996). The questionnaire consists of 26 items distributed over 4 domains: physical health, psychological health, social relations, and the environment. The responses to each question were given on a five-point Likert scale, whereby the respondents could select one of the following responses: (1) Not at all; (2) A little; (3) A moderate amount; (4) Very much; and (5) An extreme amount. The reliability and validity of the questionnaire were confirmed in the studies of Jude and Abdel (2009) and Kalfoss et al. (2021). The reliability and validity of the Serbian version of the WHOQoL questionnaire were determined by Ač-Nikolić et al. (2010) on a population of individuals over the age of 60. The research results indicated that the WHOQOL–BREF questionnaire (the Serbian version) is a valid and reliable instrument for the evaluation of quality of life among the elderly.

#### Data Analysis

For each of the applied variables of physical activity, quality of life, and BMI, descriptive statistics parameters were calculated: the means (Mean), standard deviation (Std. Dev.). Pearson’s correlation analysis was used to explore bivariate relationships between PA, BMI, and QoL. Multiple linear regression was used to estimate the relationship between PA, BMI, and QoL domains. Two models were examined for each statistical analysis. Model A was adjusted for PA only, and model B was further adjusted by including BMI in the regression model. All the data were processed using the statistical package SPSS 20.0 (SPSS Inc., Chicago, IL, United States). The significance was set at the 0.05 level.

## Results

Table 1 shows the basic parameters of descriptive statistics. Based on the average BMI values, it can be stated that the respondents belong to the group of older women who are overweight.

**TABLE 1:** Basic descriptive statistic parameters (means ± standard deviation).

**BMI**	26.1 ± 4.1	**Physical health**	23.7 ± 2.9
**PA at work**	827.5 ± 1932.3	**Psychological status**	20.2 ± 4.1
**PA in transportation**	579.5 ± 832.9	**Social relations**	9.7 ± 1.8
**PA in housework**	2924.3 ± 2235.6	**Environment**	27.3 ± 4.6
**Leisure time PA**	886.4 ± 1451.7		
**Walking**	1024.3 ± 1481.8		
**Moderate PA**	2895.4 ± 2332.7		
**High intensity PA**	1296.8 ± 1389.9		
**Total PA**	5264.7 ± 3504.7		

*PA, physical activity; Mean, mean value; Std.Dev., standard deviation.*

Table 2 shows the values of the Pearson’s coefficients of correlation for BMI and physical activity in relation to indicators of quality of life. Positive correlations (Table 2) were found between some domains of PA and Physical health and they range from 0.16 to 0.43. Statistically significant relations (*p* < 0.01) between PA and Physical health were found in Housework = 0.36, Leisure time PA = 0.27, Walking = 0.24, Moderate PA 0.43, Total PA = 0.43, while the correlations between the variables at the significance level of 0.05 were determined in PA in transportation = 0.19 and High-intensity PA = 0.16.

**TABLE 2:** Pearson correlation (r) between PA level, BMI and QoL.

	Physical health	Psychological status	Social relations	Environment
BMI	0.10	–0.04	0.04	0.05
PA at work	0.11	0.08	0.17*	–0.04
PA in transportation	0.19*	0.15	0.06	0.02
PA in housework	0.36**	0.00	–0.01	0.03
Leisure time PA	0.27**	0.02	0.06	0.26
Walking	0.24**	0.13	0.09	–0.06
Moderate PA	0.43**	0.06	0.11	0.05
High intensity PA	0.16*	–0.03	0.04	0.03
Total PA	0.43**	0.08	0.13	0.04

PA, physical activity. *p < 0.05; **p < 0.01.

The relations between some domains of PA and Social relations are significant only in PA at work (0.17, *P* < 0.05). Significant positive relationships have been found to indicate that QoL also increases with increasing PA. PA and Psychological status, as well as PA and Environment, do not correlate significantly in any domain.

Multiple linear regression analysis of different levels of PA as independent variables on individual domains of QoL (Table 3, model A) showed that there is an association of PA and Physical health (*F* = 35.93 Sig. = 0.000), more precisely, Total PA is statistically the most closely related to Physical health (β = 0.435, *t* = 5.995, Sig = 0.000), and then follows PA at work and Social relations (*F* = 4,464, β = 0.168, *t* = 2.113, Sig = 0.036).

**TABLE 3:** Multiple linear regression models between PA, BMI and domain QoL.

	Model A				Model B			
	Physical health	Psychological status	Social relations	Environment	Physical health	Psychological status	Social relations	Environment
BMI	NS	NS	NS	NS	NS	NS	NS	β = 0.263 Sig = 0.001
PA at work	NS	NS	β = 0.168 Sig = 0.036	NS	NS	NS	β = 0.168 Sig = 0.036	NS
PA in transportation	NS	NS	NS	NS	NS	NS	NS	NS
PA in housework	NS	NS	NS	NS	NS	NS	NS	NS
Leisure time PA	NS	NS	NS	NS	NS	NS	NS	NS
Walking	NS	NS	NS	NS	NS	NS	NS	NS
Moderate PA	NS	NS	NS	NS	NS	NS	NS	NS
High intensity PA	NS	NS	NS	NS	NS	NS	NS	NS
Total PA	β = 0.435 Sig = 0.000	NS	NS	NS	β = 0.435 Sig = 0.000	NS	NS	NS

*PA, physical activity; NS, not significant.*

Similar results were obtained when BMI is added to model A (Table 3, model B). In contrast to model A, model B shows statistically significant association between PA and BMI and Environment (*F* = 11,448, β = 0.263, *t* = 3.383, Sig = 0.001).

## Discussion

The current study aimed to determine the association between the level of physical activity and BMI, and quality of life among elderly women.

Based on the parameters of descriptive statistics, it could be concluded that the level of physical activity among elderly women corresponds to the level of physical activity of similar samples in other studies (Pucci et al., 2012; Puciato et al., 2017), especially when it comes to moderate physical activity.

By analyzing the results, it could be concluded that there is a statistically significant association between the aforementioned parameters and quality of life, but only in the domains of physical activity. These results are congruent with those of existing studies which focused on the correlation between physical activity and quality of life (Morimoto et al., 2006; Dugan et al., 2009; Ekwall et al., 2009; Bernard et al., 2018; Delle Fave et al., 2018; Nawrocka et al., 2019; Schultchen et al., 2019).

The results showed a statistically significant association between total physical activity, high- and moderate-intensity physical activity with the physical health of older women, where moderate PA showed higher significance levels (*p* = 0.01). Similar results were obtained by Battaglia et al. (2016) who determined the effects of adapted physical activity on the psycho-physical health of elderly women. Moderate-intensity physical activity has an advantage over high-intensity physical activity in the population of elderly women. The same results were obtained in the study of Fox et al. (2007). This study points out the benefits of physical activity for quality of life in the physical and psychological domain of women taking part in moderate-intensity physical activity. On the contrary, the study by Morimoto et al. (2006) shows that women who take part in high-intensity physical activity (>1,000 kcal/week) scored better results in all domains of quality of life, and the same results were determined in the study by Puciato et al. (2017). The reason for the different obtained results can be found in the age of the participants. The studies in which high-intensity physical activity was proven to be beneficial for the various domains of quality of life all involved younger participants. When it comes to the elderly (60+, 65+ years), the benefits of moderate-intensity physical activity are greater than those of high-intensity physical activity for physical health. Certain studies do not recommend high-intensity physical activity for the elderly (McPhee et al., 2016), especially if the remaining part of the day is marked by sedentary behavior. In addition, there are also gender differences when it comes to choosing the type of physical activity. Men are more likely to choose high-intensity activities (Hunt et al., 2014), such as sports games, while women are more likely to take part in moderate-intensity exercise, such as walking (Kassavou et al., 2013). Our results showed significant association between walking and physical health. Since walking is an activity of moderate intensity and is positively related to the quality of life, it is recommended as an activity for elderly women (Pucci et al., 2012), especially because involves a decrease in the risk of injury that may occur in high-intensity activities. A significant association between physical activity in transport and physical health was also noted. Physical activity in transport, such as walking or cycling, etc., has benefits for human health. People who use walking as a means of transportation can meet their daily needs for physical activity (Adams, 2010).

The results of the study indicate that physical activities in the domain of housework positively correlate with physical health. Women who perform moderate-intensity housework achieve better results in terms of physical health. These data are congruent with the results of other studies (Lawlor et al., 2002; Besson et al., 2008), which also state that there is no positive correlation between this type of activity and psychological health (Wen et al., 2013).

A positive association also exists between the domain of leisure-time physical activities and physical health. In their leisure time, older women choose activities that are not imposed on them, but cause them satisfaction and affect their overall wellbeing. Leisure-time physical activity is usually of moderate-intensity and has a positive effect on physical health. Similar results were obtained in Nakamura et al. (2014) study which investigated the same problem in respondents of both sexes.

The results of the regression analysis indicate that there is an association between total physical activity and physical health, which is also explained by the results of the Pearson’s correlation analysis. Further, the results indicate that there is an association between PA at work and social relations. The social domain of quality of life increases throughout lifespan (Sonati et al., 2011). Such a fact may be a specific characteristic of older women who are still employed and have good social relationships. Similar results were achieved by the study Sonati et al. (2011), which states that social relations are also influenced by socioeconomic status, level of education, and level of physical activity.

The significant association of BMI was determined only with environmental domain of quality of life. BMI and PA are positively related to environmental domain, which indicates that overweight and obese, in comparison to underweight and normally fed women, achieve better results in environmental domain of quality of life. This data is in accordance with the study results of Deng et al. (2014). In the current study, no association was determined between BMI and other domains of quality of life among elderly women. These data differ from the results of existing studies (McDonough et al., 2013; Busutil et al., 2017), in which a correlation was noted between BMI and mental health. The association between BMI and mental health is present in the younger and adult population, while there is no correlation in older women, i.e., as age increases, the correlation between BMI and mental health decreases (Huang et al., 2006).

## Conclusion

The aim of the current study was to determine the association between physical activity and BMI, and health-related quality of life in an elderly female population. Based on the results obtained, it can be concluded that there is significant association between the aforementioned parameters and quality of life, but only in the case of domains of physical activity, i.e., physical activity contributes to improved physical health among elderly women. The results of the current study indicate that high- and moderate-intensity physical exercise both have benefits for physical health, where moderate PA showed higher significance levels. BMI and quality of life were positively associated only in the environmental domain of quality of life, that is elderly women with higher BMI achieve better results in the domain of the environment. The obtained results can be used in practice when creating an exercise program for older women, where moderate physical activity (i.e., walking) is recommended. Physical activity is also related to the domain of social relations, and it is recommended to exercise in order to encourage those relations. Further studies should also focus on the association between other anthropological characteristics and abilities, and the parameters of quality of life among elderly women.

## Data Availability Statement

The raw data supporting the conclusions of this article will be made available by the authors, without undue reservation.

## Ethics Statement

The studies involving human participants were reviewed and approved by Ethics Committee of the Faculty of Sport and Physical Education, University of Niš. The ethics committee waived the requirement of written informed consent for participation.

## Author Contributions

AÐ was the leader of the research group that conducted the study. AÐ, DŽ, ZM, MŽ, LB, and MB contributed to the conception and design of the study. SP and MB organized the database and performed the statistical analysis and wrote the first draft of the manuscript. SP, AÐ, and ZM reviewed and edited the first draft. All authors contributed to the article and approved the submitted version.

## Conflict of Interest

The authors declare that the research was conducted in the absence of any commercial or financial relationships that could be construed as a potential conflict of interest.

## Publisher’s Note

All claims expressed in this article are solely those of the authors and do not necessarily represent those of their affiliated organizations, or those of the publisher, the editors and the reviewers. Any product that may be evaluated in this article, or claim that may be made by its manufacturer, is not guaranteed or endorsed by the publisher.
